# Natural and anthropogenic factors drive large-scale freshwater fish invasions

**DOI:** 10.1038/s41598-022-14556-5

**Published:** 2022-06-21

**Authors:** Marco Milardi, Aaron Iemma, Ian R. Waite, Anna Gavioli, Elisa Soana, Giuseppe Castaldelli

**Affiliations:** 1 Fisheries New Zealand - Tini a Tangaroa, Ministry for Primary Industries - Manatū Ahu Matua, 34-38 Bowen Street, Wellington, 6011 New Zealand; 2 WWF Trentino, Via Fratelli Bronzetti 29, 38122 Trento, Italy; 3 U.S. Geological Survey, Oregon Water Science Center, 2130 S.W. Fifth Avenue, Portland, OR 97201 USA; 4 Department of Environmental and Prevention Sciences, University of Ferrara, Via L. Borsari 46, 44121 Ferrara, Italy; 5 Present Address: Southern Indian Ocean Fisheries Agreement (SIOFA) c/o DAAF Bâtiment B Parc de la Providence, 97489 Saint-Denis Cedex, Réunion

**Keywords:** Ecology, Biodiversity, Biogeography, Freshwater ecology, Invasive species

## Abstract

We analyzed the large-scale drivers of biological invasions using freshwater fish in a Mediterranean country as a test case, and considering the contribution of single species to the overall invasion pattern. Using Boosted Regression Tree (BRT) models, variation partitioning and Redundancy Analysis (RDA), we found that human factors (especially eutrophication) and climate (especially temperature) were significant drivers of overall invasion. Geography was also relevant in BRT and RDA analysis, both at the overall invasion and the single species level. Only variation partitioning suggested that land use was the second most significant driver group, with considerable overlap between different invasion drivers and only land use and human factors standing out for single effects. There was general accordance both between different analyses, and between invasion outcomes at the overall and the species level, as most invasive species share similar ecological traits and prefer lowland river stretches. Human-mediated eutrophication was the most relevant invasion driver, but the role of geography and climate was at least equally important in explaining freshwater fish invasions. Overall, human factors were less prominent than natural factors in driving the spread and prevalence of invasion, and the species spearheading it.

## Introduction

Biological invasions are undoubtedly a major driver of a global decline in biological diversity^1^, but different views exists on the factors driving invasions themselves and thus they remain a controversial topic in ecology. Some authors attribute the decline of native biodiversity mainly to anthropogenic-driven habitat degradation, with biological invasions playing only a secondary role^2^. However, habitat degradation also favors the establishment of introduced species, and is thus a driver of biological invasions across different taxa and environments^3,4^, underlining the challenge to disentangle the multiple effects of anthropogenic pressure and biological invasions on native biodiversity.

Aquatic environments, and freshwaters in particular, are amongst the most impacted ecosystems by biological invasions^5,6^. Among freshwater environments, riverine systems have been identified as most fragile and vulnerable to invasions^7^. Biological invasions have been highlighted as one of the main drivers of the decline of freshwater fish biodiversity^8^ and functional diversity^9^, at different spatial scales.

Introduced species can become invasive only if they are able to establish (i.e. are able to naturally reproduce) and spread (i.e. colonize new habitats beyond the introduction area)^10^. Propagule pressure has been identified as one of the main factors affecting the establishment of freshwater fish species^11^, but it is often studied through proxies such as human population or economic indicators^12,13^. The latter are relevant proxies also for habitat degradation at a large scale, thus making it challenging to disentangle these factors. Species established because of habitat degradation might be limited to degraded habitats^14^, but other introduced species might also spread beyond these boundaries and into more natural habitats, depending on their ecological requirements and niche opportunities^15^. Indeed, natural factors tend to entangle with anthropogenic ones in determining invasion success^16^, and factors like temperatures^17^ or flow regime^18^ can dictate the outcomes of invasion.

As an example of the complex interplay among invasion drivers, disentangling the role of habitat fragmentation and flow stabilization on spread and invasion has required detailed data over long timespans^19^, but at different data resolution levels the effects of such drivers tend to remain controversial^20–22^. Disentangling the effects of single factors driving biological invasions remains a challenging and difficult task, but eminently relevant to understand the process of such a fundamental driver of global change, and made possible through detailed analysis techniques and datasets^23^.

We focused on the Mediterranean region to investigate the environmental drivers of biological invasions, because previous research identified a high risk to native biodiversity from biological invasions in this area^24–26^. We further selected Italy as a test area within the Mediterranean region, because its large altitudinal and latitudinal span create distinct climate gradients, its richness in native and endemic species, its regional geographical barriers and its long anthropogenic impact history. We selected freshwater fish as model taxa, because these have been highlighted as an endangered taxa in this region^27^, and because of detailed data availability at a large geographical scale^9^. We further worked only with already established introduced species, decades after the latest introduction, in order to be able to assess the long-term results of spread (and invasion).

Our study aimed to shed further light on the relevance and interplay of multiple factors in affecting the spread and invasion of freshwater fish. Since no geographically detailed data on introduction, early establishment and subsequent spread of the invasion was available, our investigation focused on the late-invasion stage. We chose our putative spatial drivers of fish invasion with guidance from previous studies that highlighted the importance of climate^28^, geography^29^, habitat fragmentation^30^ and anthropogenic pressure (in particular eutrophication)^23^ in shaping the spread of invasive freshwater species. Our study aimed to test the hypothesis that spread and biological invasions would be mainly driven by anthropogenic impact causing habitat degradation, and that natural variables would play a secondary role. Under this hypothesis, habitat degradation favors establishment of species and available degraded habitat influences their spread, so we also hypothesized that there would be a small overlap between the cumulative effects of anthropogenic impact and other invasion drivers in invaded areas.

## Results

Out of the 35 exotic species found in our dataset, the most widespread (goldfish, *Carassius auratus*) was present in 700 sites, but 7 exotic species were only found only at one site. Prevalence varied similarly, with the most prevalent species (brook trout, *Salvelinus fontinalis*) constituting on average 31.4% of the fish community biomass, where present, yet failing to rank higher because of their limited distribution, and other species (e.g., weatherfish, *Misgurnus fossilis*) constituting as low as 2.8% of the community on average, where present. Table 1 summarizes the top 10 results of our invasiveness index analysis.

**Table 1:** Top 10 most invasive species in our dataset, as defined by an invasiveness index resulting from a combination of colonization (% of total sampled sites where present) and prevalence (average relative abundance % where present), and their date of introduction.

Rank	Scientific name	Common name	Date of introduction	Invasiveness index	Colonization (% of total sampled sites with species presence)	Prevalence (average % of fish community where species present)
1	*Cyprinus carpio*	Common carp	1500*	359.60	17.50	20.55
2	*Oncorhynchus mykiss*	Rainbow trout	1880	184.91	6.21	29.77
3	*Pseudorasbora parva*	Topmouth gudgeon	1980*	172.34	18.03	9.56
4	*Carassius auratus*	Goldfish	1900*	154.46	18.50	8.35
5	*Silurus glanis*	Wels catfish	1950	150.77	8.99	16.78
6	*Rhodeus sericeus*	Amur bitterling	1990	132.90	14.62	9.09
7	*Gambusia holbrooki*	Mosquitofish	1930	76.81	5.31	14.46
8	*Lepomis gibbosus*	Pumpkinseed	1880	67.51	10.15	6.65
9	*Abramis brama*	Common bream	1970	65.02	4.78	13.59
10	*Ameiurus melas*	Black bullhead	1880	56.28	3.89	14.48

*Marks uncertain year of introduction.

**Figure 1:**
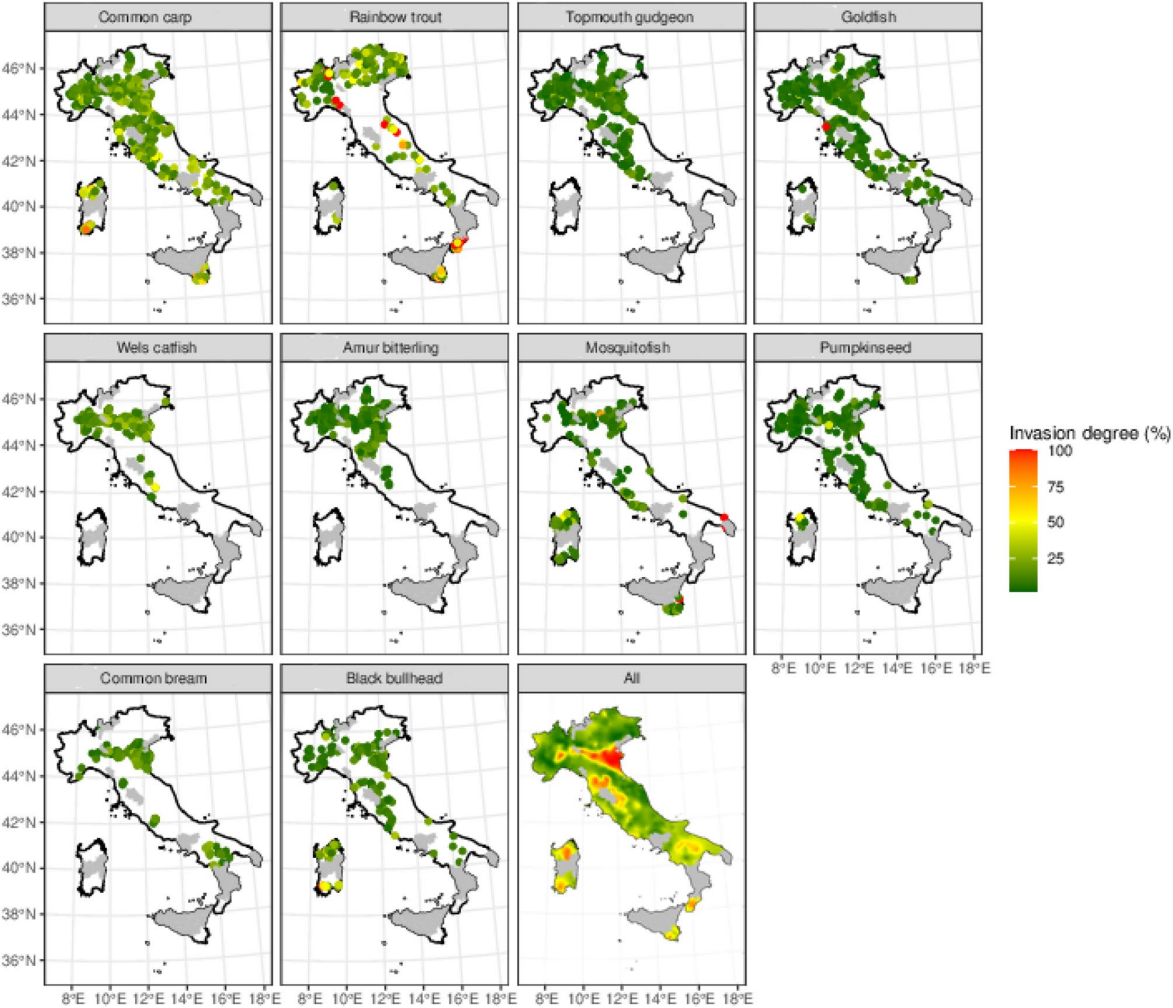
Spatial distribution of the overall invasion degree (all exotic species, last panel), and its top 10 species components (other panels, in order of invasiveness rank left to right and top to bottom), the main response variable in this study. This figure was created with R^31^.

The spatial distribution of overall invasion showed a main hotspot in the northern part of the country, in correspondence with the mid- to low stretches of the Po River, the longest in the country and flowing mainly through an alluvial floodplain (Fig. 1, last panel), but smaller hotspots of invasion were visible in the southern and central parts of the country, as well as on the islands. The distribution of species like common bream and wels catfish was close to that of the main hotspots (Fig. 1), while species like goldfish and common carp were much more widespread throughout the country (Fig. 1).

**Figure 2:**
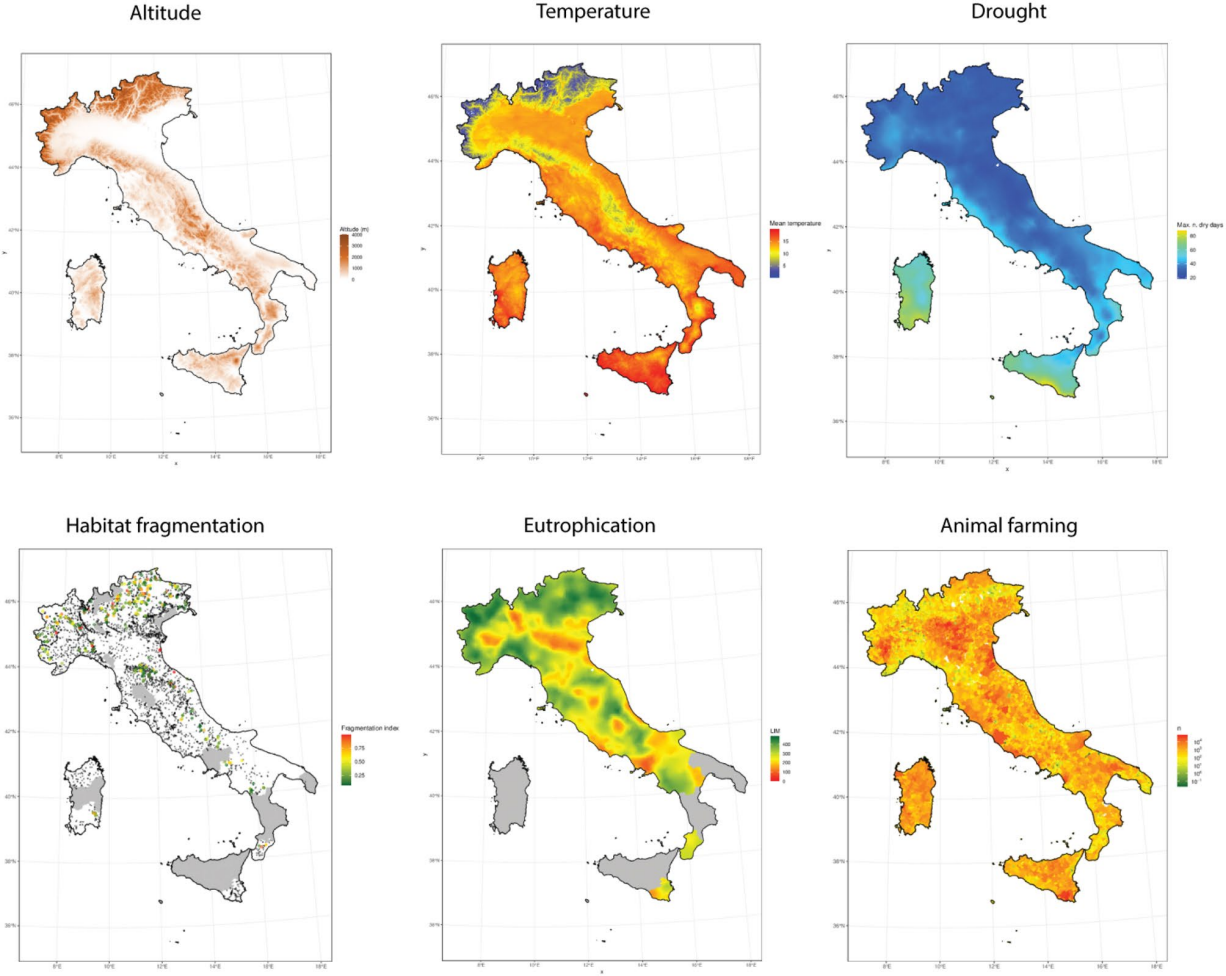
Spatial distribution of the most relevant drivers of invasion, analyzed in this study. From left to right, and top to bottom, altitude, temperature, drought period, habitat fragmentation, eutrophication and animal farming. See Supplementary Fig. 1 for the distribution of the other main drivers analyzed. This figure was created with R^31^.

Large floodplains in the study area showed higher temperatures, eutrophication and animal farming levels but lower precipitation and relatively low habitat fragmentation than higher altitude areas (Fig. 2).

**Figure 3:**
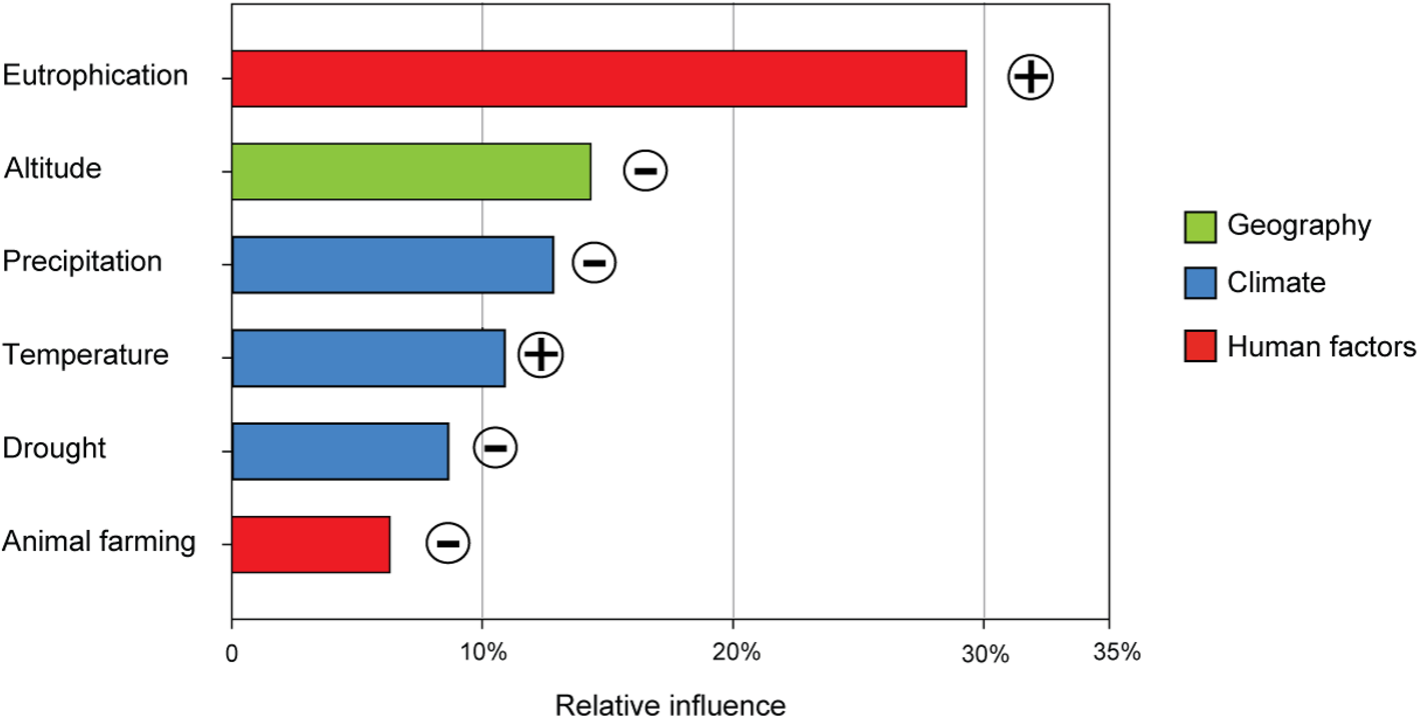
Relative influence of drivers affecting invasion degree, according to the outputs of BRT analysis. Pluses (⊕) and minuses (⊖) on the right of each bar represent the direction of the influence (positive and negative, respectively). Spatial autocorrelation explained 17.7% of the variation. See Supplementary Fig. 2 for additional details on the response variation through the gradient of each driver.

Overall invasion was fairly well-explained (CV R^2^ = 0.57) by a limited set of invasion drivers in the final BRT model. Among these, the relative influence of human factors (mainly eutrophication, at 29.3%, and animal farming, at 6.3%) was the highest (Fig. 3), with mixed effects: increasing invasion with increasing eutrophication, but decreasing invasion with increasing animal farming density. Climate factors (precipitation, temperature and drought) had a collective relative influence of 32.4% (Fig. 3), with mixed directions of influence. Geography (altitude) had a relative influence of 14.3% on the invasion, with a negative effect increasing invasion at lower altitudes (Fig. 3). Spatial autocorrelation (SAC) explained 17.7% of the overall variation.

**Figure 4:**
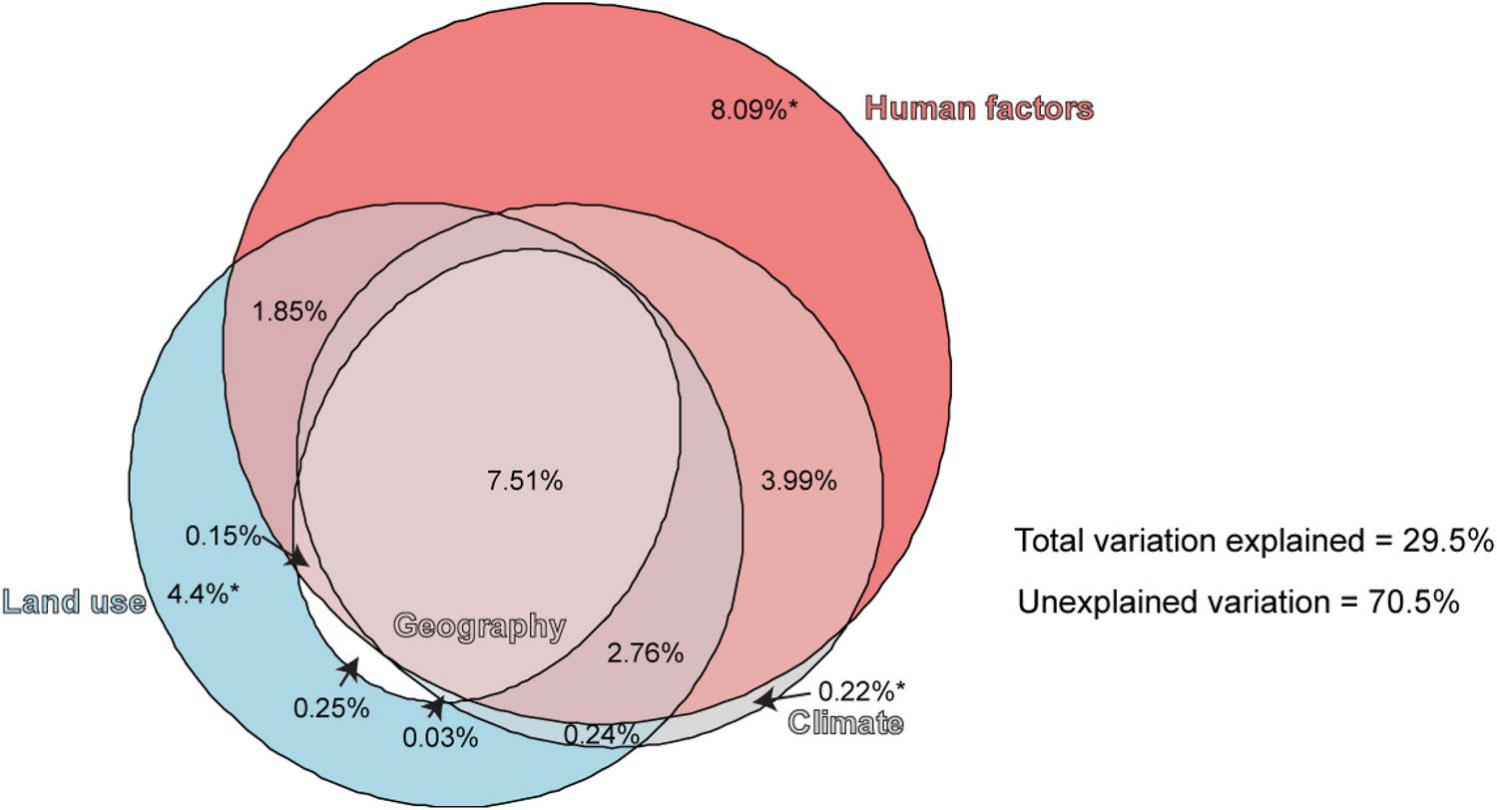
Area-proportional Euler-Venn graphs illustrating the partitioning of variation explanatory power on invasion degree among the main invasion drivers groups. *Marks significant unique variation explained (*P* < 0.01).

The variation partitioning of invasion degree delivered similar results as the BRT, with an overall explained variation of 29.5% (Fig. 4). There was a high overlap among different driver groups (7.5%), but human factors had the most relevant unique (8%) variation explained of all the variable groups (Fig. 4). Land use was the second most relevant (unique variation explained 4.4%), while Climate (unique variation explained 0.22%) and Geography were less relevant (Geography had no unique variation explained) (Fig. 4). All unique fractions were significant (*P* < 0.01).

**Figure 5:**
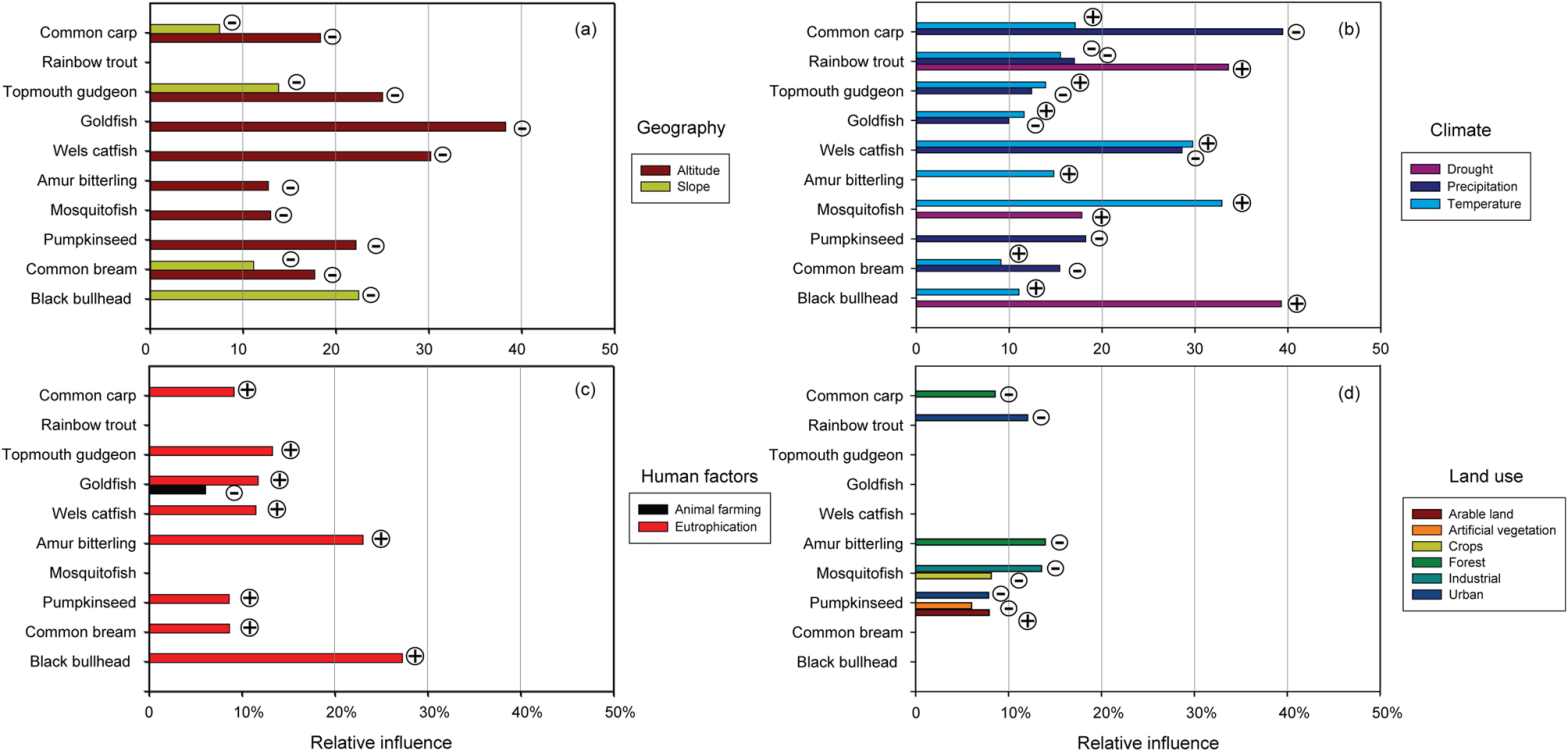
Relative influence of each driver (and drivers group) on the distribution and prevalence in the community of the top 10 invasive freshwater fish in Italy (listed in descending order of invasiveness, from the top), as derived from BRT analysis. Pluses (⊕) and minuses (⊖) on the right of each bar represent the direction of the influence (positive and negative, respectively). On average, SAC explained 28.7% of the variation for each species. See Supplementary Fig. 3 for additional details on the response variation of each species through the driver gradient.

Geography (either altitude or slope, or both), negatively influenced most of the top 10 invasive species (e.g. common bream CV R^2^ = 0.29 or wels catfish CV R^2^ = 0.35), with the exception of rainbow trout (CV R^2^ = 0.20) that didn’t show any response (Fig. 5a). Climate had a more diverse pattern, with temperature positively influencing invasion of most species (except rainbow trout), and either precipitation or drought (respectively negatively and positively) influencing most species (e.g. topmouth gudgeon CV R^2^ = 0.39, mosquitofish CV R^2^ = 0.18) (Fig. 5b). Human factors, especially eutrophication, positively influenced most species (especially amur bitterling CV R^2^ = 0.39, common carp CV R^2^ = 0.29, goldfish CV R^2^ = 0.29, and black bullhead CV R^2^ = 0.11), except topmouth gudgeon, rainbow trout and mosquitofish (Fig. 5c). Land use had the least effects, with pumpkinseed (CV R^2^ = 0.11) being the notable exception (Fig. 5d). Accounting for SAC did not improve the model fits for common carp, wels catfish and black bullhead, but slightly improved the other species’ models fit; on average, SAC explained 28.7% of the variation.

**Figure 6:**
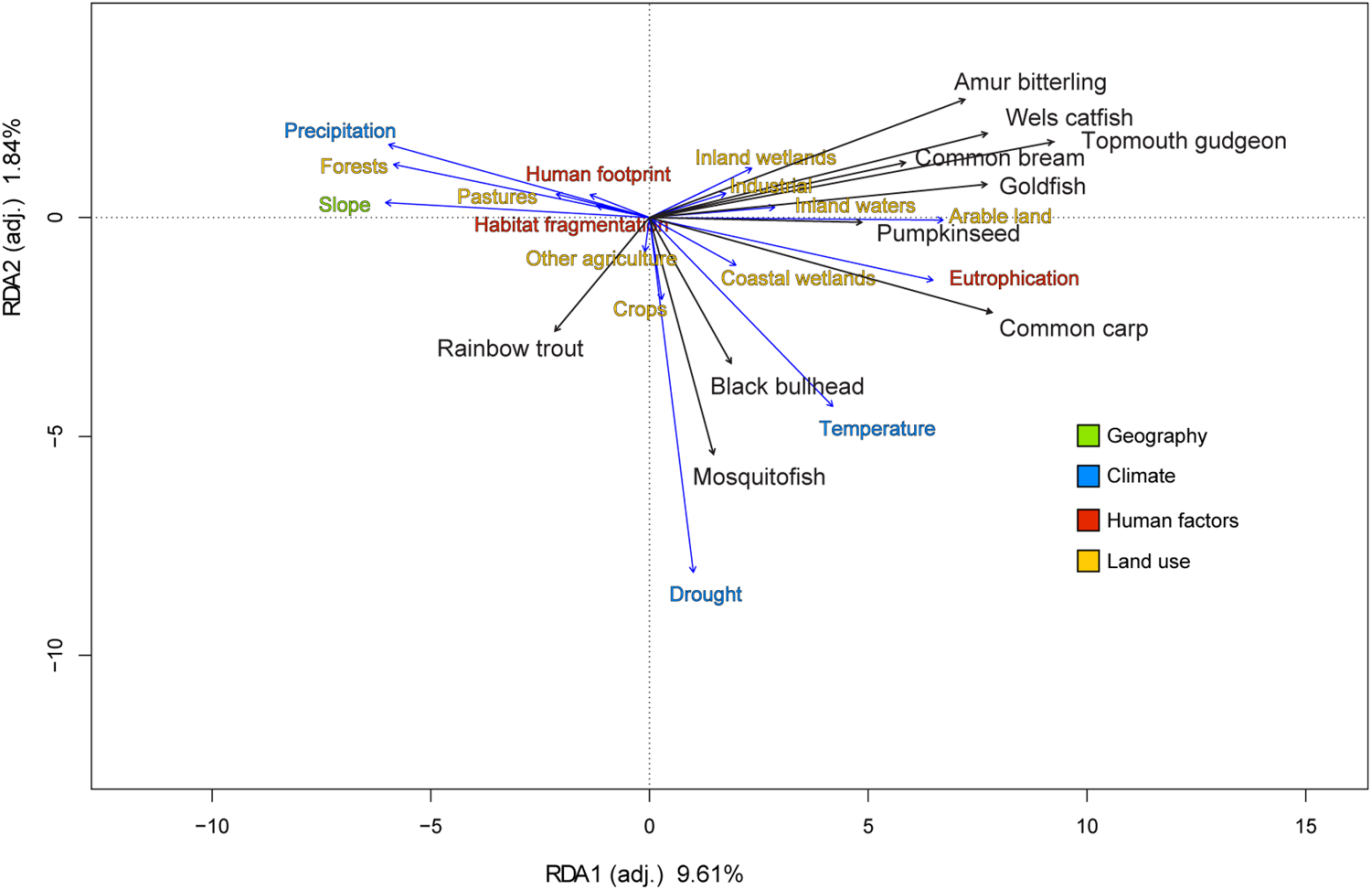
Biplot of RDA results, showing the effect of geography, climate, human factors and land use drivers on the distribution of the top 10 invasive freshwater fish species in Italy. Driver names in different colors identify different invasion drivers groups (see legend). The main axis (RDA1, horizontal) explains 9.61% of the variance (F = 432.56; *P* < 0.01), while the secondary axis (RDA2, vertical) explains 1.84% of the variance (F = 83.06; *P* < 0.01). Some variables close to origin (i.e. with negligible effects) were removed to improve readability.

The RDA analysis further underlined the difference between rainbow trout and the other 9 species, with the latter being positively influenced by low slope (collinear with altitude), eutrophication, temperature and land use (Fig. 6). High slope, high drought and precipitation seemed to drive rainbow trout, with habitat fragmentation having only a relatively minor effect (Fig. 6). Both RDA axes were significant (RDA1 F = 432.56, *P* < 0.01; RDA2 F = 83.06, *P* < 0.01), and the overall adjusted R squared was 12.41%.

## Discussion

Confirming our first hypothesis only in part, the significance of human factors (especially eutrophication) but also of climate (especially temperature) in driving overall invasion in the area was highlighted by all analyses. Geographical variables had a high relevance in BRT and RDA analysis, both at the overall and the species level, but variation partitioning underlined a different pattern, with geography having the least effects (either single or combined with other driver groups). Only variation partitioning suggested that land use was the second most significant driver group, whereas the other analyses downplayed its significance, particularly considering the species level. Also our second hypothesis was confirmed only in part, as there was considerable overlap between different invasion drivers, but with land use and human factors standing out for single effects, underlining a consistent interplay among driver groups. For the first time, we analyzed the contribution of single freshwater fish species to the overall invasion pattern at a large spatial scale in a Mediterranean country, using their detailed prevalence in the community and colonization rates to rank their invasion. In general, there was a good accordance between overall and single species invasion patterns in the BRT analysis, but the significance of human factors at the species level was downplayed, whereas that of geography and climate was emphasized, compared to the overall level. All analyses underlined that overall invasion was widespread in the lowlands, especially in the Po River plain (north of the country), which likely explains the inverse linkage with slope and altitude, as well as temperature. There was general accordance both between different analyses, and between invasion results at the overall and the species level, as most invasive species share similar ecological traits and prefer lowland river stretches. Rainbow trout was an exception, preferring cooler waters in streams and overall dryer climate zones.

Similarly to previous localized studies^23^, eutrophication was highlighted as one of the main drivers within the human factors group, but lowland waters are naturally nutrient enriched, so that the human component might act in synergy with the natural component in these areas. The human footprint index also included population density, which is often considered a proxy for human-induced eutrophication, yet human footprint had a much lower relevance than eutrophication for fish invasion, perhaps due to the mixed effects of its other components, or because linkage with eutrophication is only partially human-mediated (as clearly illustrated in Fig. 6). While they can have other types of impacts on waterways, livestock farming and agriculture in general are also considered to be important sources of eutrophication^32^. Yet their effects were marginal on fish communities in our study area, and sometimes apparently contradictory, e.g. in the case of goldfish where animal farming had an opposite effect compared to eutrophication. Despite land use being highlighted as the second most important driver by the variation partitioning and RDA analysis, the BRT analysis did not select land use as a relevant factor for overall invasion, and showed a moderate response to land use only for a small subset of the most invasive species (e.g. pumpkinseed). Nevertheless, as a general pattern, anthropic land use positively drove the invasion, whereas forests or more natural land cover had the opposite effect. This was most likely linked to the distribution of most invasive species in the lowlands, however, rainbow trout was distributed at higher altitudes and thus positively related to natural forest land cover.

We did not include geographic coordinates in our analyses, as they are usually considered a proxy for more detailed variables that we analyzed directly. However, our preliminary analysis indicated that the invasion increased with increasing northern latitude, which is in line with broad ecological analyses for these latitudes^33^. Altitude (and consequently slope) was instead one of the main factors driving overall invasion, as well as the invasion of several of the most invasive species, further underlining the distinction between lowlands and higher elevations. However, a major factor regulating invasion location could be the ecological traits of introduced species: in our case most introduced species (with very few exceptions) were not adapted to streams, and were thus less able to colonize high-elevation habitats. Elevation is also a good proxy for temperature, even if differences could be less marked in running waters, which suggests that temperature could be one of the factors leading to invasion.

Temperature, defining areas with slightly different climate over the long term, was the most relevant climate driver of invasion, with higher invasion in warmer areas, lending further credit to the hypothesis that temperature tolerance is an advantageous trait for invasive freshwater fish in this area^34^. The Supplementary Figs. 2 and 3 illustrate that temperature responses of invasive species are bell shaped around an “optimum”, coherent with ecological theory. However, the effects of temperature on freshwater invasion are still controversial^34,35^, and might be dependent on local factors. Further analyses on native species could reveal different temperature optima, perhaps underlining different abilities to tolerate warmer climates under current climate prediction scenarios, and lead to the conclusion that climate is a key invasion driver. However, such analyses should also investigate climate trends, preferably using water temperature data, as these might reveal that temperature has not significantly changed when considering long timespans^34^, but particular attention needs to be invested in both analysis techniques^35^ and sampling location to avoid overgeneralizing.

Habitat fragmentation is one of the main human impacts in mountain areas, where the gravitational gradient allows more energetically-convenient hydropower but also where overall invasion was lower, and mainly driven by rainbow trout. Habitat fragmentation was lowest in the lowlands, but there might be areas (e.g. agricultural areas with extensive canal networks) where watercourse fragmentation was under-detected, due to the challenges to artificial intelligence and human operators in the detection of smaller barriers. To date and to our knowledge, this is the only assessment of river fragmentation at the national scale, but it can be further improved e.g. possibly through citizen science projects reporting the presence of barriers through mobile applications (Barrier Tracker app, https://portal.amber.international). Surprisingly our analysis did not identify habitat fragmentation as a relevant driver of invasion, contrarily to recent findings in other areas^19^, perhaps because the effects of flow regulation and habitat fragmentation on invasion can be counterintuitive see e.g.^21,36^, and our analysis mainly accounted for migration barriers, rather than flow effects. Furthermore, fish stocking for recreational fisheries could be a stronger driver than habitat fragmentation for some species distribution (e.g. rainbow trout). Recent research showed that most rivers are naturally fragmented through an intermittent hydrological regime^37^, and that climate variables are good proxies for flow regime. Cumulative precipitation, and drought, in our data were characteristic of Mediterranean climates, and show a high degree of variability throughout the area, with precipitation being higher on the mountains and drought being more pronounced in lowlands, southern areas, and islands. Because lowland areas receive less precipitation, most of the invasive species were linked to lower levels of rain, but also lower levels of drought, as most of the invaded sites were located in the northern plains where precipitation is relatively low but also relatively frequent through the year. Drought partly explained the distribution of species like mosquitofish and rainbow trout, which were common also in the southern parts of the country and the islands, but was otherwise a weak predictor of invasion. It could be hypothesized that Mediterranean ecosystems and native species are naturally resilient to highly intermittent flow conditions and drought typical of this region, and that fewer introduced species would be likewise adapted to invade such environments, whereas flow stabilization could lead to more invasion^19^. Flow stabilization might have further contributed to invasion in the lowlands, downstream from the areas with high fragmentation, but further data and analyses would be needed to investigate its role in the observed invasion pattern.

The temporal and spatial mismatch between environmental and biotic variables, given the nearly impossible task to monitor all variables at a similar temporal and spatial resolution, is a common problem of studies in this field^38^. While perhaps unavoidable, temporal and spatial mismatches can be minimized, using improved interpolation and minimal extrapolation, and considering timescales comparable to species assemblages turnover time^29,39^. More detailed spatial and temporal information on introduction history could provide information to further interpret the patterns observed. As a n example, mosquitofish was widely introduced to reduce malaria in wetlands between 1920 and 1940s, without knowledge on its effectiveness for this purpose^40^, nor on its detrimental competition with endemic and endangered killifish^41^. As a result, their invasion distributions perhaps are the result of spread and subsequent selection by unsuitable habitat. Habitat-induced selection requires at least decadal timescales^42^, and involve other factors such as species origin, invasiveness potential and overall available habitat^43^, which could partially explain why no clear patterns were immediately evident (see Table 1) between time since introduction and invasiveness rank and confirming that most introduced species in our study are in late invasion stages. It is perhaps worthy to note that species invasiveness can vary through time, so that species that ranked high in our analysis could have been either more or less invasive at different invasion stages, including the current situation. Furthermore, invasiveness can be defined based on impacts, which are not directly assessed in our ranking. Developing taxonomical knowledge and techniques (leading to new definitions of species), and population dynamics (often poorly known), further complicate any assessment of invasiveness through time.

Contrarily to most other taxa, freshwater fish exhibit high rates of voluntary introductions (e.g. to enhance sport fisheries) and human-mediated spread be a relevant factor in species invasion^12^. The European Union developed an international strategic framework for invasive species management^44^, which also allows for national declinations. Pumpkinseed is the only invasive fish species of concern across EU states, and its distribution and prevalence here is more detailed than what is currently published^45^. Our work provides a clear evidence-based ranking of the top invasive freshwater fish species, which could serve as a basis for further impact assessments and the development of national priority lists. National legislation approved in the late 90 s to regulate introductions of exotic species (DPR 357/97) has been ineffective, and recent attempts to apply it more strictly to recreational fisheries (DM 2/4/2020) have been stalled, thus calling into question the strategic policy aimed at limiting freshwater fish invasion.

Our analyses revealed a large overlap between different invasion drivers, shedding further light on the importance of each driver in favoring the invasion. While most of our analyses underlined human-mediated eutrophication as the most relevant invasion driver, the role of geography and climate was at least equally important in explaining freshwater fish invasions. These results provide a new perspective on the invasion mechanisms, where natural factors are as prominent as human factors in driving the spread and prevalence of invasion and the species spearheading it. In this perspective, policies aiming at reducing anthropogenic impacts (such as the ones in place to reduce human nutrient pollution) could only partly address freshwater fish invasion, and should further consider regional differences in natural factors. Of course, preventing the introduction of new species should always be prioritized as it is the most cost-effective management strategy^46^ and renaturalization of sites should not be discouraged, but the latter might not be necessary to control invasions if effective population control or harvest management strategies are found.

## Materials and methods

### Invasion

We used freshwater fish biodiversity data collated by and described in Milardi, et al.^47^. In summary, the dataset included 3777 sites sampled 1999–2014, recorded a total of 99 different fish species (35 of which were exotic and already established, even if some are restricted to areas with thermal springs), spanned > 11 degrees of longitude (~ 1200 km) and 10 degrees of latitude (~ 1100 km), covering streams at altitudes -2.7–2500 m above sea level. Community turnover was not a relevant factor in our study, because fish communities are typically stable over these timescales and the data was collected in a restricted timeframe within each area^29,39^. Furthermore, time elapsed since last introductions was sufficient to analyze distribution patterns after major invasions had already occurred see e.g.^23,48^.

Abundance of each species sampled during the monitoring was recorded with Moyle classes (Moyle and Nichols, 1973), which were weighted according to body-size classes in order to obtain a body-mass-corrected abundance, hereafter referred to simply as abundance. We then calculated an invasion degree, i.e. the share of introduced species in freshwater fish communities, based on the abundance of introduced and native species see e.g.^9,49^. A high invasion degree equals to a high share of introduced species and a low share of native species.

We also selected the top 10 invasive species as further response variables, under the assumption that these would be the main components of the invasion degree, but would respond to different invasion drivers based on each species’ ecology. Invasiveness rank was defined through an index obtained by multiplying colonization (share of sites colonized) and prevalence (average relative abundance in the fish community) of each introduced species. The relative abundance of each of these species in the fish community was used as a response variable, being a measure comparable to invasion degree for single species.

### Invasion drivers

We tested a combination of geographical, climate and anthropogenic impact factors as potential drivers of invasion. To avoid temporal mismatches, we chose time periods that overlapped as much as possible with our biological data.

We used basin area, altitude and slope (derived from a seamless digital elevation model of the whole Italian territory at 10 m resolution, Tarquini, et al.^50^) as geographical variables.

We derived climate data from available series of long-term national monitoring (http://www.scia.isprambiente.it/). We used daily air temperature (2000–2009), measured at a total of 2266 sites throughout the country, as a proxy for temperature regimes. We also used cumulated annual precipitations, number of annual dry days (precipitation < 1 mm) and maximum number of consecutive dry days (all from 2000–2009), measured at a total of 3098 sites throughout the country, as a proxy for hydrological regimes.

We used an index the 2009 Human Footprint^51^, based on eight variables (built-up environments, population density, electric power infrastructure, crop lands, pasture lands, roads, railways, and navigable waterways), as a proxy for overall anthropogenic impact and propagule pressure. The lower the proxy, the smaller the anthropogenic impact. We also further explored single components of anthropogenic impact, by analyzing separately variables related to land use in 2012 (Copernicus Land Monitoring Service—https://land.copernicus.eu/pan-european/corine-land-cover/).

We further used an index see e.g.^52^, based on the concentration of 7 different parameters linked to nutrient levels (oxygen saturation, biochemical oxygen demand, chemical oxygen demand, NH_4_, NO_3_, total P and *E. coli* levels), measured at monthly intervals 2006–2010, at 1636 sites throughout the country, as a proxy for eutrophication levels. High proxy values correspond to low eutrophication levels. We further added the intensity of animal farming in 2010 (numbers of animals reared, ISTAT—http://dati.istat.it/Index.aspx?DataSetCode=DCSP_CONSISTENZE).

Finally, we used the presence of migration barriers as a proxy for riverine habitat fragmentation. We detected migration barrier locations through high-resolution cloud-free satellite images, and manually classified them in 4 categories (small jump, high jump, minor dam, major dam) according to their type and size (as gauged from visual characteristics, e.g. the presence of upstream retention basins).

### Estimating invasion drivers at fish sampling points

We derived elevation of each fish sampling point from the DEM, which we similarly used to calculate the total area of the basin above the site, cropped at a 10 km distance from the fish sampling point. In lowland areas, where basin determinations were uncertain due to low elevation gradients, we derived the same variables from an area of 10 km radius around the sampling point. We derived the slope using a 10 km long river network segment, centered on the fish sampling point.

We used daily temperatures, cumulated annual precipitations, number of annual dry days, and maximum number of consecutive dry days, to build mean integrated nested Laplace approximated (INLA) annual layers for the decade 2000–2009^53^. We assigned to each fish sampling point the value of the mean interpolated in a 5 km radius around the point (temperature regime proxy) or the mean over the basin above the sampling point (hydrological regime proxy).

We calculated the minimum, maximum, sum and mean values of the overall anthropogenic impact proxy and expressed them as densities in the cropped basin above the sampling point. We used a 10 km cutoff under the assumption that it would capture the most relevant pressures for any given sampling point, that other pressures further upstream would be less relevant as their pressure would be partly dampened by environmental buffers and avoid overlap between different sampling points along the same watercourse. We used a similar calculation for the density of animal farming (number of poultry, sheep, pigs and cattle), which were converted in livestock units (poultry = 0.01, cattle = 1, sheep = 0.1, pigs = 0.5) so that they could be combined into one variable. Similarly, we used the percentage of each land cover class in the cropped basin, aggregated at the highest level (i.e. Artificial Surfaces, Agricultural areas, Forest and semi-natural areas, Wetlands, Water bodies).

We calculated the eutrophication proxy as a mean of INLA-interpolated annual layers and projected these over the river network. We then used the mean (and relative SD) of the proxy using a 10 km long river network segment, centered on the fish sampling point.

For habitat fragmentation we used three variables: the number of reachable barriers along the river network, the mean distance of reachable barriers, and a habitat fragmentation index. This index used reachable barriers only, and was built as:

1 $$\frac{1}{{\frac{\pi }{2}}}\tan \left( {\frac{1}{barrier \,distance} \times barrier \,category } \right)$$

To vary non-linearly between 0 (least fragmented) and 1 (most fragmented). We chose a 10 km cutoff for these variables, as it was in line with other measures and the average distance most freshwater fish species are expected to move up or downstream^54^, recognizing that some species have both shorter and longer migration spans.

### Data analysis

After a preliminary analysis we retained the following variables for a full analysis and grouped them in 4 large invasion drivers groups: Geography (slope, altitude), Climate (mean values of temperature and precipitation, as well as mean maximum number of dry days (drought)), Human factors (mean densities of human footprint and livestock units, mean eutrophication index and habitat fragmentation index) and Land use (percentages of broad land cover classes). We used mean densities, rather than absolute values, trying to reduce any area-dependent effects.

We developed Booster Regression Tree (BRT) models for both invasion degree and the top 10 invasive taxa. BRT models are among a family of techniques used to advance single-classification or regression trees by combining the results of sequentially fit regression trees to reduce predictive error and improve overall performance^55–57^. BRT models have been shown to have superior performance over linear modeling techniques especially with data that are often highly skewed, such as environmental data^55,58^, and are considered an efficient method to describe non-linear relationships between variables and automatically incorporate interactions between variables. We reduced explanatory variables in each final BRT model by using a combination of variable importance (VI) scores, evaluation of interactions, and partial dependency responses (see below) following the approach outlined by Elith, et al.^55^ to minimize overfitting. All variables with VI < 7 were deleted and the remaining variables were used to develop the final BRT model. Calculations of VI values are based on the number of times a variable is selected for splitting, weighted by the squared improvement to the models as a result of each split, averaged over all trees. After a first run, we used the BRT analysis residuals to test for spatial autocorrelation (SAC) through the Moran’s I test and, where SAC was found, we built a SAC autocovariate that was fed into the model to account for SAC. Final model choice relied on best model fit, and residuals were tested to confirm that spatial autocorrelation was reduced. The relative importance of each variable is scaled so that the sum adds to 100, with higher numbers indicating stronger influence on the modeled response. When two variables that we interpreted as explaining the same type of variation within the same stressor type, and showing the same type of response pattern, occurred in the top 10 most important variables, we kept only one variable in the final model, unless dropping one of the variables reduced the CV R^2^ (cross-validation R^2^) beyond a reasonable level, based on expert judgement. We used CV R^2^ (cross validation) values instead of R^2^ to compare performance among BRT models because CV R^2^ values are more conservative and less likely to be inflated by potential overfitting. We calculated CV R^2^ values by holding 25% (bag fraction) of the sites out for each regression tree split, then used the withheld sites to test the percentage of deviance explained by the split^55^. We used partial dependency plots to visualize the direction of individual drivers effects on the response variable, after accounting for the average effects of all other explanatory variables in each final model^55,56^. A partial dependency plot is a scatter plot of an individual driver vs biotic metric and the modeled response form for that metric, where the response curves indicate the general shape, direction, and potential breakpoints (i.e., effect levels) for each driver. We ran Moran’s I test and built a SAC autocovariate using a Voroni tessellation with the functions of the spdep package^59^, including testing for negative SAC. We ran BRT models using the gbm package^60,61^ and specific code from Elith, et al.^55^. Because Elith et al.’s code optimizes the number of trees run in each model, the number of trees can vary for each model; however, all models had at least 1000 trees.

We investigated the collinearity of variables through the variance inflation factor (VIF) within each variable group, and we excluded collinear variables (VIF > 8) from variation partitioning and RDA analyses. We performed variation partitioning through a partial regression to find the relative contributions of each group of invasion drivers (i.e., Geography, Climate, Human factors and Land use) in explaining invasion degree. The total variation was thus partitioned into different components: the variance explained by a single group of explanatory variables, the variance jointly explained by variables of two or three groups and unexplained variance (Legendre & Legendre, 2012). The significance of unique fractions was tested using permutation-based ANOVA with 999 permutations^62^. Geography (slope), Climate (mean temperature, mean precipitation, mean drought), Human factors (human footprint, animal farming, eutrophication, river fragmentation) and Land use (all land use subclasses) were ultimately retained. We used the varpart function of the “vegan” R package^62^ to partition the variance, and the “eulerr” R package^63^ to represent the outputs through area-proportional Euler-Venn diagrams.

We also used Redundancy Analysis (RDA) to investigate the variation of the top 10 invasive species explained by invasion drivers^64^, using adjusted R^2^ values to report the variance explained. We used the RDA function of the “vegan” R package^62^ to run this analysis and test the significance of axes using permutation-based ANOVA with 499 permutations.

All analyses were performed in R^31^.

## Supplementary Information

Supplementary Figures.

## Data Availability

Underlying data for this paper belongs to local institutions and is already available in the public domain.
