# Supplementary material for: Natural and anthropogenic factors drive large-scale freshwater fish invasions
Source: Sci Rep. 2022 Jun 21;12:10465. doi: 10.1038/s41598-022-14556-5 (PMC9213492; doi:10.1038/s41598-022-14556-5)
Supplement: Supplementary file 1 — Supplementary Figures. [file 41598_2022_14556_MOESM1_ESM.docx]

**Supplementary Material**


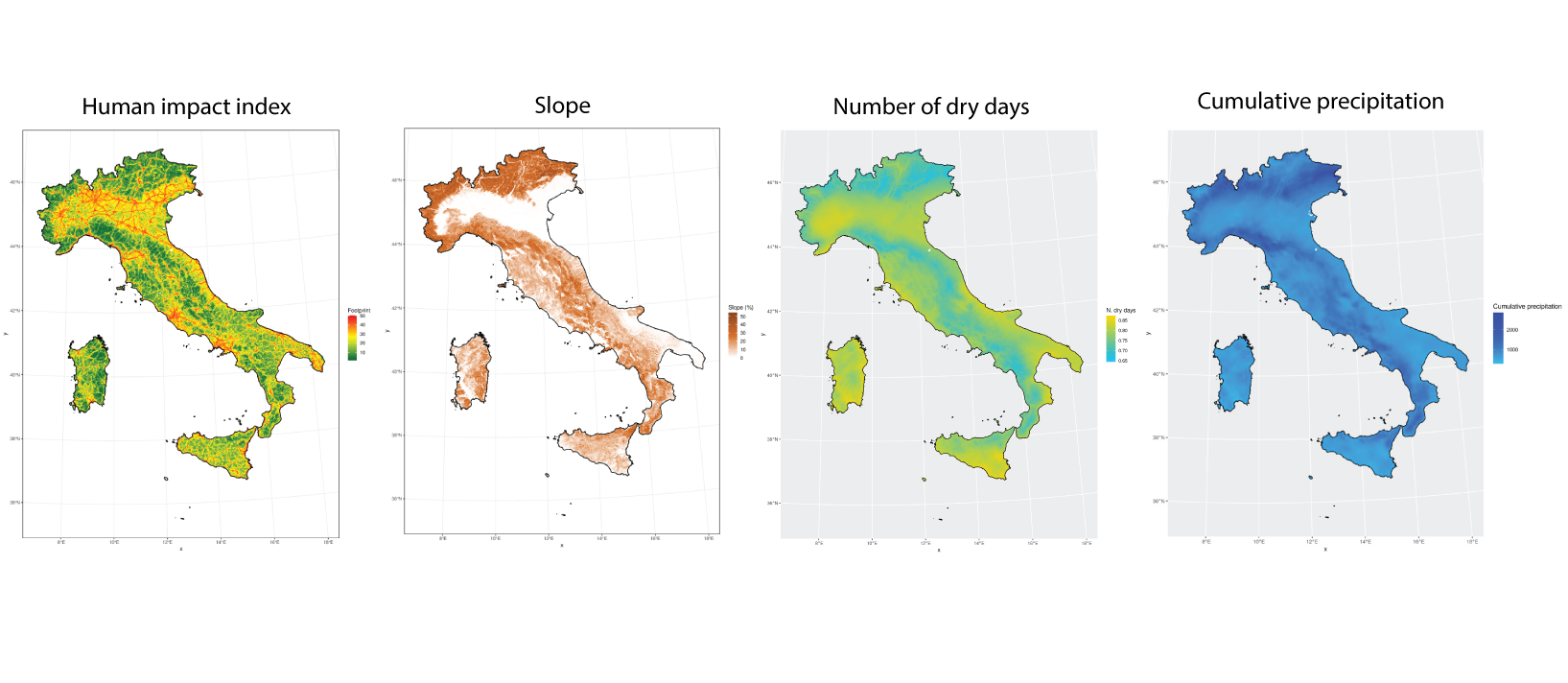


Supplementary Figure 1 – Spatial distribution of the other relevant drivers of invasion, analyzed in this study. From left to right, human impact index, slope, number of dry days and cumulative precipitation. This figure was created with R [^28^](#_ENREF_28).


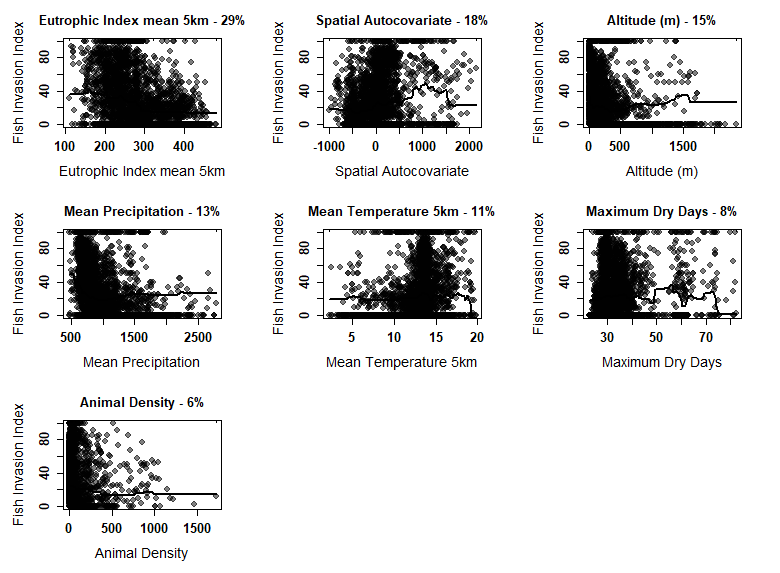


Supplementary Figure 2 – Partial dependency plots describing invasion degree response to the 6 most relevant invasion drivers, as derived from BRT analysis, including spatial autocorrelation (SAC). Eutrophication is lower at higher values of the index.


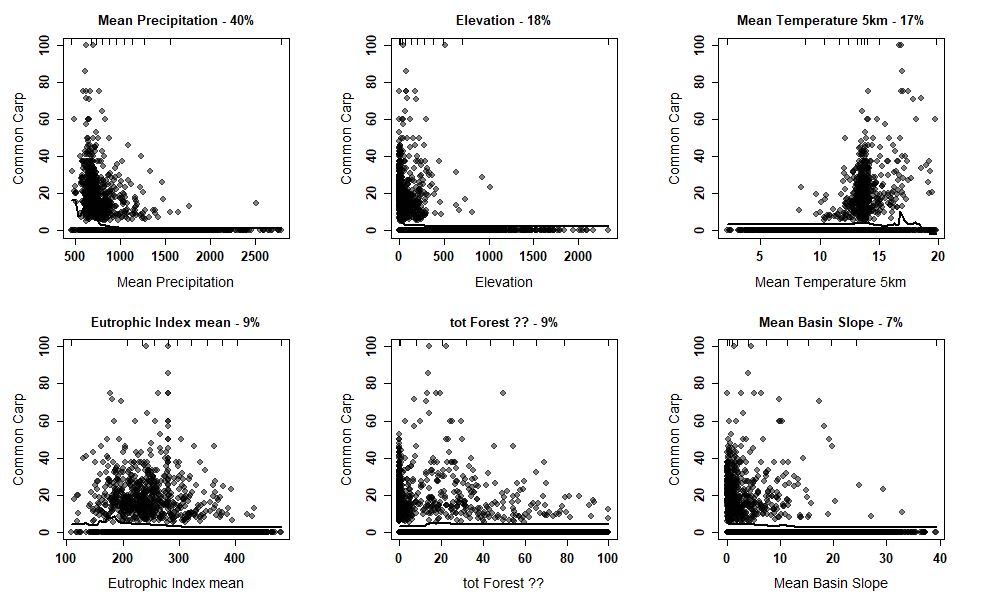


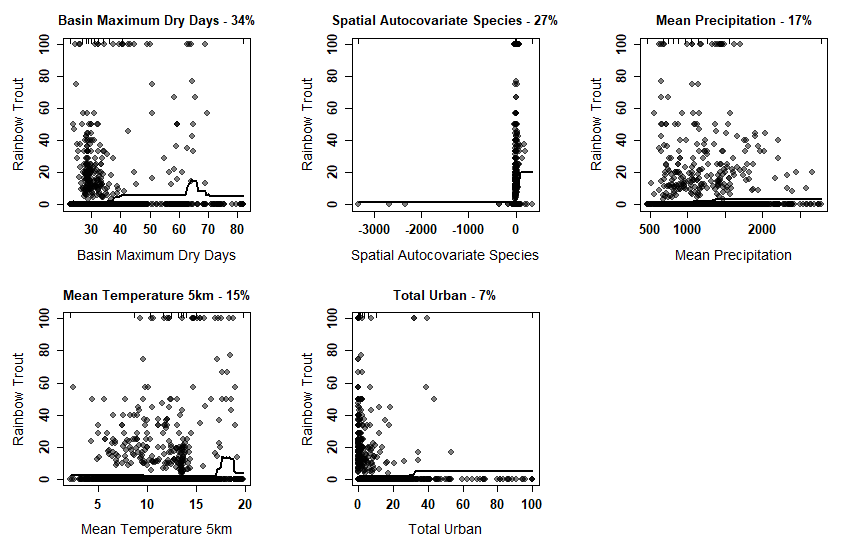


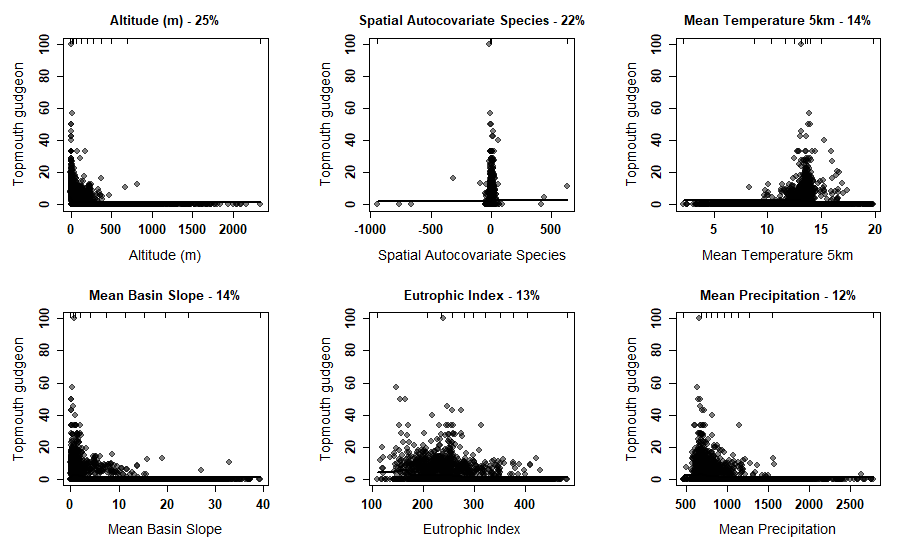


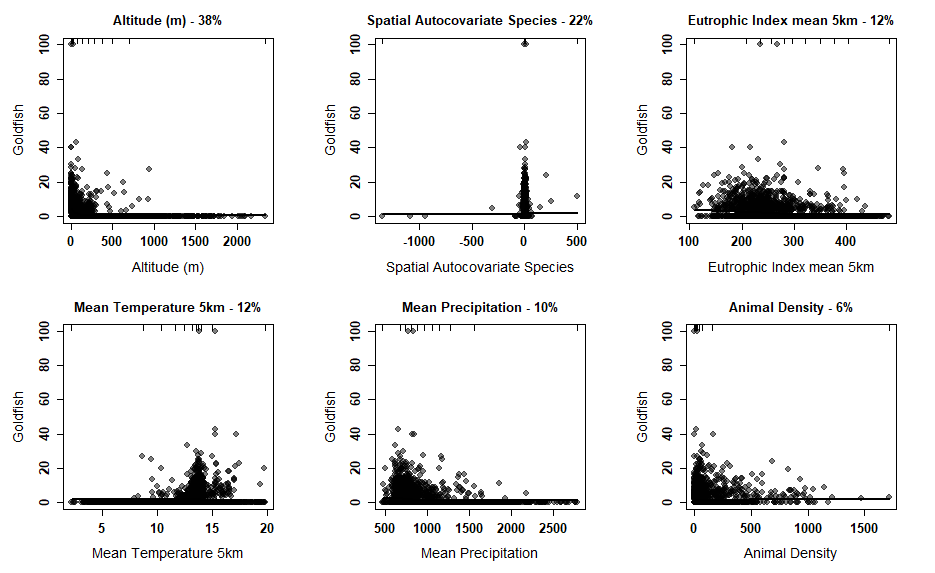


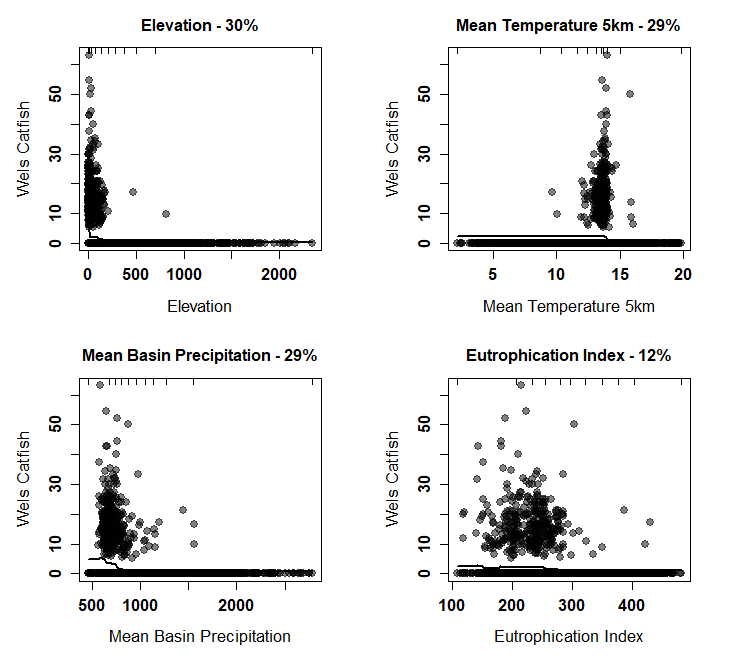


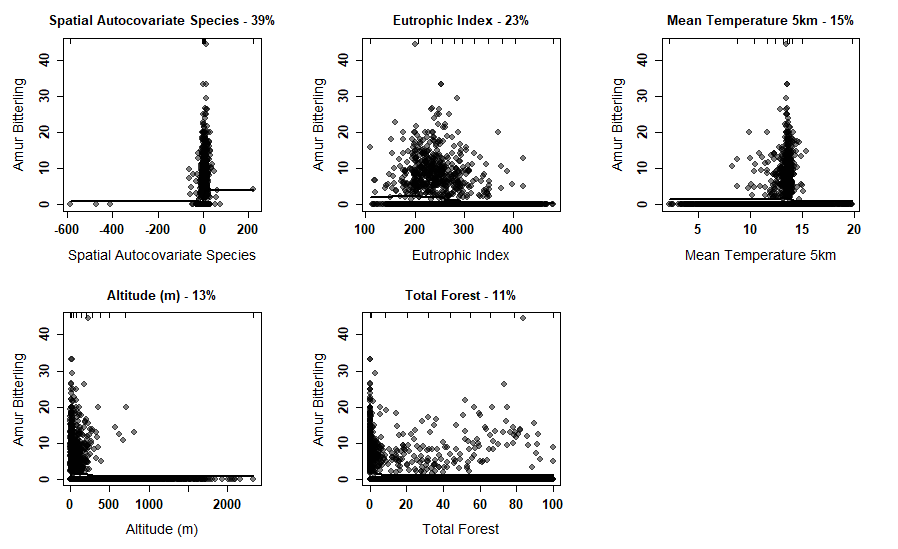


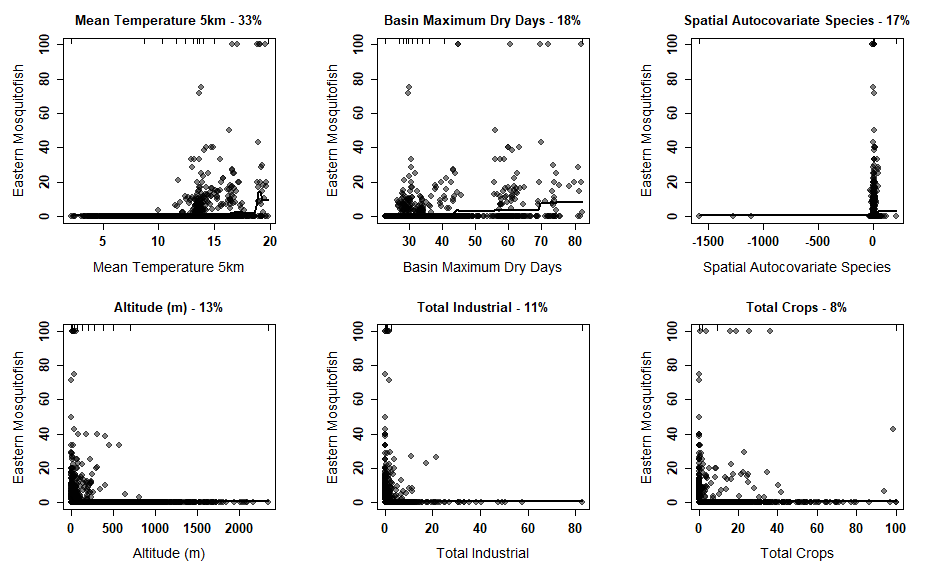


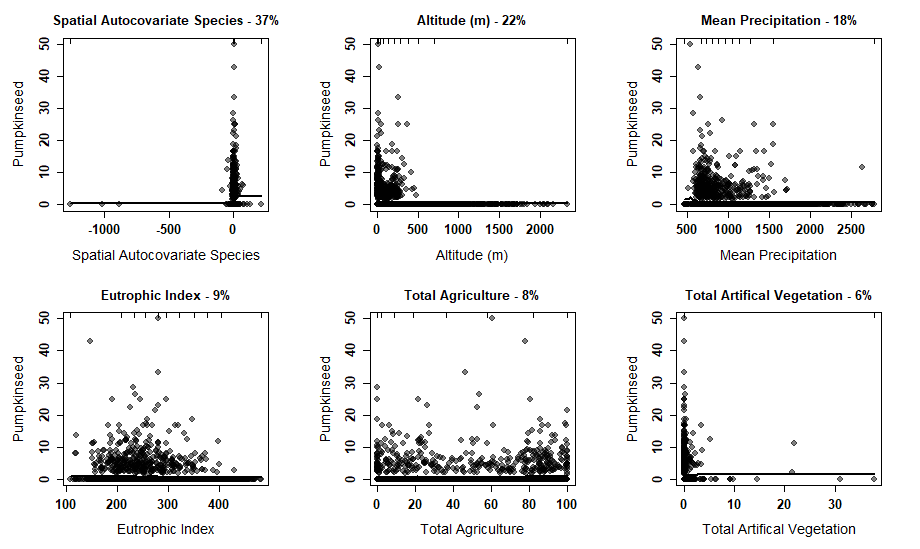


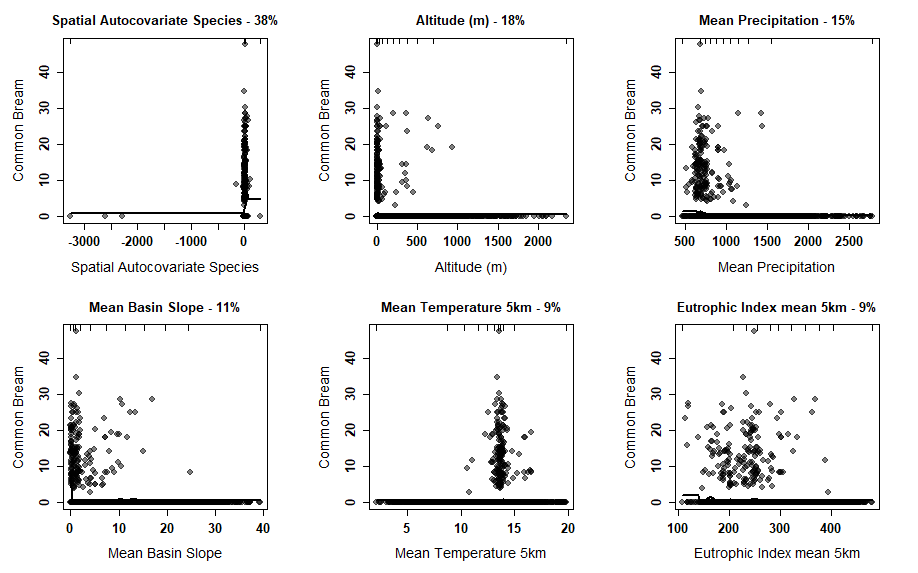


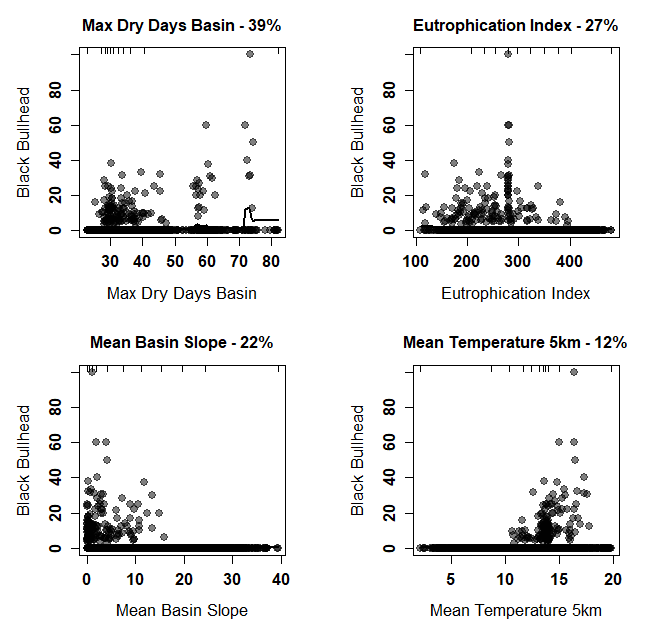


Supplementary Figure 3 – Partial dependency plots describing prevalence response of the top 10 most invasive freshwater fish species to the most relevant invasion drivers, as derived from BRT analysis. Eutrophication is lower at higher values of the index. Plots are listed in descending order of invasiveness, from most invasive to least invasive, and species common names are indicated in each vertical axis.
